# Long-term outcomes of esophageal and gastric cancer patients with cardiovascular and metabolic diseases: A two-center propensity score-matched cohort study

**DOI:** 10.2478/jtim-2023-0112

**Published:** 2023-09-02

**Authors:** Bo Zhou, Zhixin Wang, Qifeng Dou, Wenbin Li, Yangyang Li, Zhengqiang Yan, Peisheng Sun, Baosheng Zhao, Xiumin Li, Fangfang Shen, Bangjie Zhang, Mingzhou Guo

**Affiliations:** Department of Gastroenterology and Hepatology, Chinese PLA General Hospital, Beijing 100853, China; Department of Gastroenterology and Hepatology, the First Medical Center, The Third Affiliated Hospital of Xinxiang Medical University, Xinxiang 453003, Henan Province, China; Department of Urology Surgery, The First Affiliated Hospital of Xinxiang Medical University, Xinxiang 453199, Henan Province, China; Department of Cardiovascular Surgery, Beijing Anzhen Hospital affiliated to Capital Medical University, Beijing 100029, China; Department of Cardiovascular Surgery, Henan Provincial Chest Hospital, Zhengzhou 450008, Henan Province, China; Department of General Surgery, The First Affiliated Hospital of Xinxiang Medical University, Xinxiang 453199, Henan Province, China; Department of Thoracic Surgery, The First Affiliated Hospital of Xinxiang Medical University, Xinxiang 453199, Henan Province, China; Henan Key Laboratory of Tumor Molecular Therapy Medicine, Xinxiang Medical University, Xinxiang 453003, Henan Province, China; The Key Laboratory for Tumor Translational Medicine, The Third Affiliated Hospital of Xinxiang Medical University, Xinxiang 453003, Henan Province, China

**Keywords:** long-term outcome, gastric cancer, esophageal cancer, cardiovascular and metabolic diseases, propensity score matching

## Abstract

**Background and Objectives:**

An increased risk of cardiovascular and metabolic diseases (CVMDs) among patients with cancer suggests a potential link between CVMD and cancer. The impact of CVMD on the survival time of patients with esophageal and gastric cancer remains unknown. We aimed to determine the incidence of CVMD and its impact on the longterm outcomes in esophageal and gastric cancer patients.

**Methods:**

A total of 2074 cancer patients were enrolled from January 1, 2007 to December 31, 2017 in two hospitals, including 1205 cases of esophageal cancer and 869 cases of gastric cancer, who were followed up for a median of 79.8 and 79.3 months, respectively. Survival time was analyzed using the Kaplan–Meier method before and after propensity score matching.

**Results:**

The incidence of CVMD in patients with esophageal and gastric cancer was 34.1% (411/1205) and 34.3% (298/869), respectively. The effects of hypertension, diabetes, and stroke on the long-term survival of esophageal and gastric cancer patients were not significant (all *P *> 0.05). The survival time was significantly longer in esophageal cancer patients without ischemic heart disease than in patients with ischemic heart disease, both before matching (36.5 *vs.* 29.1 months, *P *= 0.027) and after matching (37.4 *vs.* 27.9 months, *P *= 0.011). The survival time in gastric cancer patients without ischemic heart disease was significantly longer than in patients with ischemic heart disease, both before (28.4 *vs.*17.5 months, *P* = 0.032) and after matching (29.5 *vs.*17.5 months, *P *= 0.02).

**Conclusion:**

The survival time of esophageal and gastric cancer patients with ischemic heart disease was significantly reduced compared to that of esophageal and gastric cancer patients without ischemic heart disease.

## Introduction

Cardiovascular and metabolic diseases (CVMDs) and cancer are the top two causes of death worldwide.^[1, 2, 3, 4]^ There is growing concern regarding the impact of CVMDs on the long-term prognosis of cancer patients, as the incidence of CVMDs has been reported to be increasing in cancer patients.^[5, 6, 7]^ In 2020, esophageal and gastric cancer-related deaths accounted for more than one-tenth of cancer-related deaths, becoming the sixth and third leading causes of cancer-related deaths worldwide, respectively.^[8,9]^ Previous studies have revealed that the risk of CVMDs is higher in patients with cancer (including breast cancer, Hodgkin lymphoma, leukemia, non-Hodgkin lymphoma, and lung cancer) than in noncancerous controls.^[10, 11, 12]^ A recent report suggested that 18.0% of cancer patients had at least one type of CVMD, and the most common CVMD among all cancer patients was hypertension, followed by diabetes, stroke, and ischemic heart disease.^[13]^ Although cumulative studies have revealed an increased risk of CVMDs in cancer patients, the impact of CVMDs on the survival time of cancer patients remains unclear. More evidence is needed to clarify the association between CVMDs and survival time in cancer patients, as comorbidity with cancer and CVMDs are usually excluded from clinical trials and most studies have focused on cancer-specific prognoses. Both tumors and CVMDs may influence the overall survival (OS) time of patients with cancer. Thus, it is desirable to collect and analyze the detailed clinical factors in esophageal and gastric cancer patients with CVMDs and to determine the impact of CVMDs on the long-term outcomes of these patients. To this end, clinical information on esophageal and gastric cancers from two hospitals in Xinxiang, a high-incidence area of esophageal and gastric cancer, was collected and analyzed using a pseudo-randomized method with propensity score matching.

## Methods

### Patients and study design

This cohort included patients diagnosed with esophageal and gastric cancer at the First Affiliated Hospital of Xinxiang Medical College and the Third Affiliated Hospital of Xinxiang Medical College, China. Data were collected retrospectively from January 1, 2007 to December 31, 2017. The inclusion criteria were as follows: (1) newly diagnosed primary esophageal and gastric cancers, (2) available detailed diagnostic and treatment records in these hospitals, (3) age > 20 years, and (4) no history of other malignancies. The exclusion criteria were as follows: (1) lack of follow-up data, (2) unknown therapeutics, and (3) incomplete clinical information. The protocol was approved by the institutional review boards of the participating hospitals. All procedures were performed in accordance with the Declaration of Helsinki. Informed written consent was obtained from all patients before inclusion in the study.

### Data collection

The esophageal and gastric cancer cohorts were followed up. Patient information included age, sex, smoking history, drinking history, family history of tumors, relapse, lymph node metastasis at diagnosis, distant metastasis at diagnosis, tumor stage, tumor differentiation, treatment regimen, and survival time. The tumor stage was determined according to the 8th American Joint Committee on Cancer classification system.^[14]^ Detailed information on CVMDs of patients was also collected, including hypertension, diabetes, ischemic heart disease, and stroke. The International Classification of Diseases standard ICD-10 was applied to these four diseases.

### Follow-up

The follow-up data were based on medical records from these hospitals and phone call interviews. Follow-up was conducted once a year and ended in December 2021. OS was calculated from the time of diagnosis to the date of death or end of the follow-up.

### Statistical analysis

We divided all cancer patients into two groups: those with and without CVMD. Statistical analysis was performed using the chi-squared test or Fisher’s exact test for categorical variables. To better determine the impact of CVMDs on cancer patient survival and reduce the interference of other factors, we performed a propensity score matching analysis between the two groups. Propensity score matching was performed using a 1:1 nearest-neighbor matching algorithm with a 0.02 caliper to minimize selection bias. A logistic regression propensity score model for the presence of CVMD was created, and the following variables were included in the propensity score model: age, sex, smoking history, drinking history, family tumor history, relapse, lymph node metastasis at diagnosis, distant metastasis at diagnosis, tumor stage, tumor differentiation, and treatment regimen. Absolute standardized differences (ASDs) of covariates before and after matching were calculated to evaluate propensity score matching effectiveness. Covariables were considered well balanced if the ASD was < 10%.^[15, 16, 17]^ The Kaplan–Meier method was used to estimate the OS between the two groups, and the statistical significance of differences was assessed using the log-rank test. *P* < 0.05 was considered statistically significant. All statistical analyses were performed using Stata 15.1 (Stata Corp., College Station, TX, USA) and Statistical Package for the Social Sciences (SPSS) 26.0 (IBM Corp., Armonk, NY, USA).

## Results

### Baseline and clinicopathological characteristics

**Figure 1:**
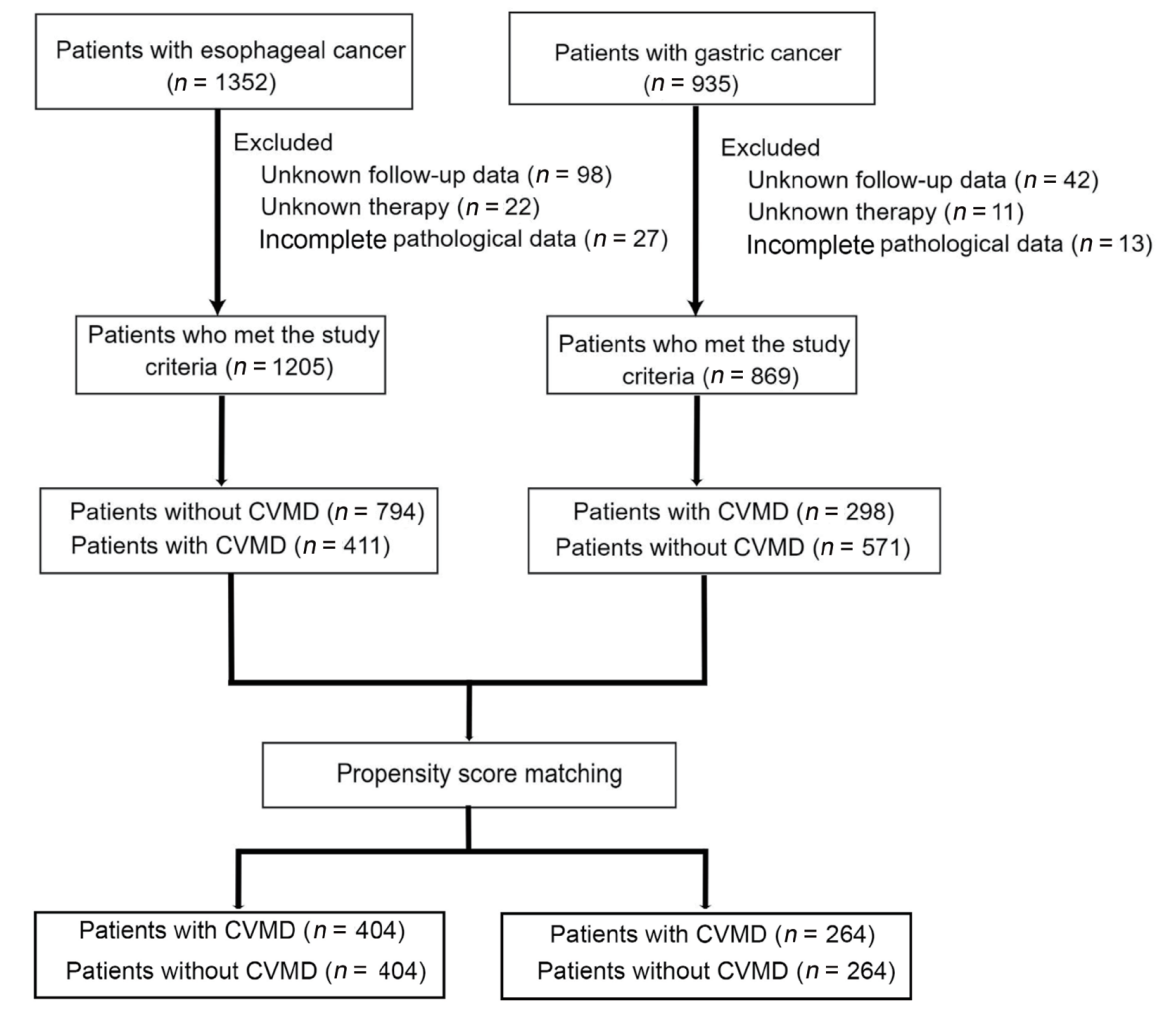
Diagram of the study design. CVMDs: cardiovascular and metabolic diseases.

Based on the inclusion criteria, 1352 patients with esophageal cancer and 935 patients with gastric cancer were enrolled in the study. Among these, 147 esophageal cancer patients and 66 gastric cancer patients were excluded due to a lack of follow-up data, unknown treatment regimens, or incomplete clinicopathological data, resulting in 1205 esophageal cancer patients and 869 gastric cancer patients eligible for this study (Figure 1). All patients with esophageal and gastric cancer were divided into groups: those with and without CVMD. Patients in the groups with CVMD were matched individually to those in the groups without CVMD using propensity scores. Consequently, 404 pairs of matched esophageal cancer patients and 264 pairs of matched gastric cancer patients were created.

**Figure 2:**
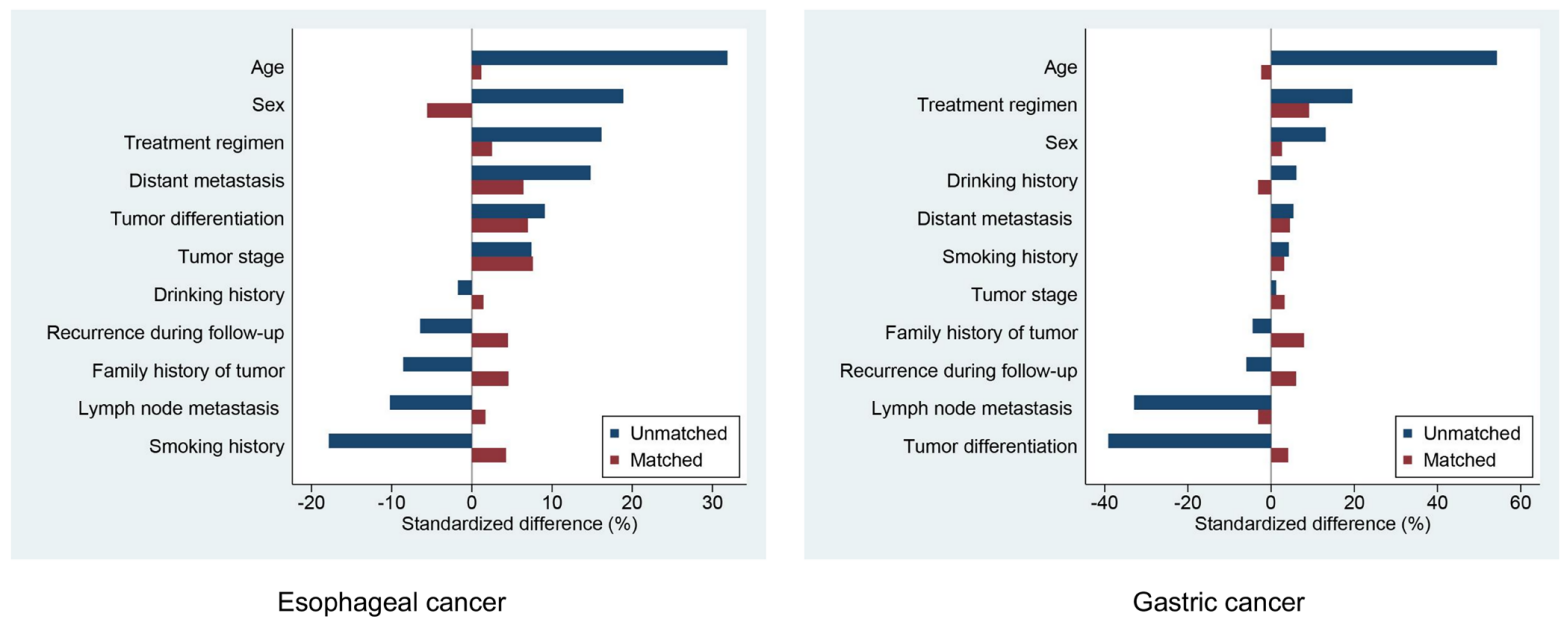
Standardized differences in baseline characteristics between cancer patients with and without CVMD, before and after propensity score matching. CVMDs: cardiovascular and metabolic diseases.

The baseline characteristics of the overall and propensity score-matched esophageal cancer patient cohorts are shown in Table 1. Of these patients, 733 (60.8%) were men, 343 (28.5%) were aged < 60 years, 680 (56.4%) were aged 60–75 years, and 182 (15.1%) were aged > 75 years. Sex, age, smoking history, distant metastasis, and treatment regimens were significantly different between the two groups before matching. No significant differences in baseline characteristics were found among the 404 matched pairs of patients with esophageal cancer. In 404 pairs of propensity score-matched patients, the standardized differences for all covariates were < 10%, suggesting a substantial balance between the two groups (Figure 2).

**Table 1:** Baseline characteristics of the overall and propensity score-matched esophageal cancer patient cohorts

**Characteristics**	**Before matching (*n* = 1205)**			**After matching (*n* = 808)**		
	**Patients without CVMD (*n* = 794)**	**Patients with CVMD (*n* = 411)**	***P* value**	**Patients without CVMD (*n* = 404)**	**Patients with CVMD (*n* = 404)**	***P* value**
Sex			0.002			0.437
Male	508 (64.0)	225 (54.7)		214 (53.0)	225 (55.7)	
Female	286 (36.0)	186 (45.3)		190 (47.0)	179 (44.3)	
Age			< 0.001			0.471
≤ 60 years	263 (33.1)	80 (19.5)		90 (22.3)	80 (19.8)	
61–74 years	429 (54.0)	251 (61.0)		234 (57.9)	251 (62.1)	
≥ 75 years	102 (12.9)	80 (19.5)		80 (19.8)	73 (18.1)	
Smoking history			0.004			0.526
No	510 (64.2)	298 (72.5)		299 (74.0)	291 (72.0)	
Yes	284 (35.8)	113 (27.5)		105 (26.0)	113 (28.0)	
Drinking history			0.775			0.836
No	683 (86.0)	356 (86.6)		351 (86.9)	349 (86.4)	
Yes	111 (14.0)	55 (13.4)		53 (13.1)	55 (13.6)	
Family history of tumor			0.162			0.504
No	577 (72.7)	314 (76.4)		315 (78.0)	307 (76.0)	
Yes	217 (27.3)	97 (23.6)		89 (22.0)	97 (24.0)	
Recurrence during follow-up			0.296			0.485
No	722 (90.9)	381 (92.7)		379 (93.8)	374 (92.6)	
Yes	72 (9.1)	30 (7.3)		25 (6.2)	30 (7.4)	
Lymph node metastasis			0.096			0.806
No	565 (71.2)	311 (75.7)		307 (76.0)	304 (75.2)	
Yes	229 (28.8)	100 (24.3)		97 (24.0)	100 (24.8)	
Distant metastasis			0.013			0.37
No	673 (84.8)	325 (79.1)		332 (82.2)	322 (79.7)	
Yes	121 (15.2)	86 (20.9)		72 (17.8)	82 (20.3)	
Tumor stage			0.058			0.769
I	338 (42.6)	176 (42.8)		185 (45.8)	173 (42.8)	
II	200 (25.2)	90 (21.9)		91 (22.5)	90 (22.3)	
III	135 (17.0)	59 (14.4)		56 (13.9)	59 (14.6)	
IV	121 (15.2)	86 (20.9)		72 (17.8)	82 (20.3)	
Tumor differentiation			0.267			0.606
Well differentiated	379 (47.7)	176 (42.8)		189 (46.8)	176 (43.6)	
Moderately differentiated	281 (35.4)	158 (38.5)		149 (36.9)	154 (38.1)	
Poorly differentiated	134 (16.9)	77 (18.7)		66 (16.3)	74 (18.3)	
Treatment regimen			0.008			0.723
Without chemo-radiotherapy	492 (62.0)	222 (54.0)		227 (56.2)	222 (55.0)	
With chemo-radiotherapy	302 (38.0)	189 (46.0)		177 (43.8)	182 (45.0)	

Data are presented as *n* (%). CVMDs: cardiovascular and metabolic diseases.

Table 2 shows the baseline characteristics of the patients with gastric cancer before and after propensity score matching. Of the patients with gastric cancer, 651 (74.9%) were men, 371 (42.7%) were aged < 60 years, 399 (45.9%) were aged 60–75 years, and 99 (11.4%) were aged > 75 years. Age, lymph node metastasis, tumor differentiation, and treatment regimens were significantly different between the two groups before matching. None of the factors were significantly different among the 264 matched pairs of gastric cancer patients. After matching, the standardized differences for all measured covariates were < 10%, suggesting a good balance between the two groups (Figure 2).

**Table 2:** Baseline characteristics of the overall and propensity score-matched gastric cancer patient cohorts

**Characteristics**	**Before matching (*n* = 869)**			**After matching (*n* = 528)**		
	**Patients without CVMD (*n* = 571)**	**Patients with CVMD (*n* = 298)**	***P* value**	**Patients without CVMD (*n* = 264)**	**Patients with CVMD (*n* = 264)**	***P* value**
Sex			0.064			0.768
Male	439 (76.9)	212 (71.1)		195 (73.9)	192 (72.7)	
Female	132 (23.1)	86 (28.9)		69 (26.1)	72 (27.3)	
Age			< 0.001			0.073
≤ 60 years	298 (52.2)	73 (24.5)		83 (31.4)	73 (27.7)	
61–74 years	222 (38.9)	177 (59.4)		137 (51.9)	161 (61.0)	
≥ 75 years	51 (8.9)	48 (16.1)		44 (16.7)	30 (11.3)	
Smoking history			0.55			0.719
No	370 (64.8)	187 (62.8)		168 (63.6)	164 (62.1)	
Yes	201 (35.2)	111 (37.2)		96 (36.4)	100 (37.9)	
Drinking history			0.391			0.727
No	486 (85.1)	247 (82.9)		218 (82.6)	221 (83.7)	
Yes	85 (14.9)	51 (17.1)		46 (17.4)	43 (16.3)	
Family history of tumor			0.537			0.349
No	426 (74.6)	228 (76.5)		209 (79.2)	200 (75.8)	
Yes	145 (25.4)	70 (23.5)		55 (20.8)	64 (24.2)	
Recurrence during follow-up			0.414			0.452
No	528 (92.5)	280 (94.0)		251 (95.1)	247 (93.6)	
Yes	43 (7.5)	18 (6.0)		13 (4.9)	17 (6.4)	
Lymph node metastasis			< 0.001			0.725
No	258 (45.2)	183 (61.4)		150 (56.8)	154 (58.3)	
Yes	313 (54.8)	115 (38.6)		114 (43.2)	110 (41.7)	
Distant metastasis			0.44			0.559
No	557 (97.5)	288 (96.6)		259 (98.1)	257 (97.3)	
Yes	14 (2.5)	10 (3.4)		5 (1.9)	7 (2.7)	
Tumor stage			0.279			0.233
I	120 (21)	72 (24.1)		56 (21.2)	65 (24.6)	
II	230 (40.2)	101 (33.9)		118 (44.7)	95 (36.0)	
III	207 (36.3)	115 (38.6)		85 (32.2)	97 (36.7)	
IV	14 (2.5)	10 (3.4)		5 (1.9)	7 (2.7)	
Tumor differentiation			< 0.001			0.838
Well differentiated	66 (11.6)	53 (17.8)		45 (17.1)	40 (15.1)	
Moderately differentiated	267 (46.8)	178 (59.7)		154 (58.3)	157 (59.5)	
Poorly differentiated	238 (41.6)	67 (22.5)		65 (24.6)	67 (25.4)	
Treatment regimen			0.005			0.302
Without chemo-radiotherapy	466 (81.6)	219 (73.5)		208 (78.8)	198 (75.0)	
With chemo-radiotherapy	105 (18.4)	79 (26.5)		56 (21.2)	66 (25.0)	

Data are presented as *n* (%). CVMDs: cardiovascular and metabolic diseases.

### OS in esophageal and gastric cancer patients with and without CVMD

For esophageal cancer patients, the 5-year OS rate was 39% before matching, during a median follow-up period of 79.8 (interquartile range [IQR] 69.9–91.4) months. In addition, the 5-year OS rates in the groups with and without CVMD were 38% and 40%, respectively. For gastric cancer patients, the 5-year OS rate was 43% before propensity score matching, during a median follow-up period of 79.3 (IQR 67.6–92.5) months. The 5-year OS rates in the groups with and without CVMD were 40% and 45%, respectively.

In esophageal cancer patients, the median OS of groups without and with CVMD was 37.2 and 33.8 months, respectively, before matching (*P *= 0.41) and 39.9 and 33.8 months, respectively, after matching (*P *= 0.46). OS was not significantly different between the groups without CVMD and with CVMD before and after matching (Supplementary Figure 1). In the groups of gastric cancer patients without and with CVMD, the median OS was 27.8 and 27.4 months, respectively, before matching (*P *= 0.62) and 28.7 and 27.9 months, respectively, after matching (*P *= 0.57). The survival times of the two groups were not significantly different (Supplementary Figure 1).

### Characteristics of CVMDs in esophageal and gastric cancer patients

The constitution of CVMDs in patients with esophageal and gastric cancer is shown in Supplementary Table 1. Common CVMDs included ischemic heart disease, hypertension, diabetes, and stroke. The prevalence of CVMDs in patients with esophageal cancer was 34.1% (411/1205), among which the prevalence of hypertension, diabetes, ischemic heart disease, and stroke was 22.7% (273/1205), 8.1% (98/1205), 8% (96/1205), and 6.9% (83/1205), respectively. The incidence of one, two, and multiple CVMDs in patients with esophageal cancer was 24.3% (293/1205), 8.2% (99/1205), and 1.6% (19/1205), respectively. The incidence was 34.3% (298/869) in gastric cancer patients, among which the prevalence of hypertension, diabetes, ischemic heart disease, and stroke was 24.5% (213/869), 6.8% (59/869), 7.2% (63/869), and 7.5% (65/869), respectively. The incidence for gastric cancer patients with one, two, and multiple CVMDs was 24.7% (215/869), 7.5% (65/869), and 2.1% (18/869), respectively. The median OS was 37.2 *versus* 36 *versus* 30.3 *versus* 32.4 months (*P* = 0.052) before matching and 39.9 *versus* 35.7 *versus* 30 *versus* 33.3 months (*P *= 0.081) after matching in esophageal cancer patients without and with one, two, or multiple subtypes of CVMDs, respectively (Supplementary Figure 2). OS was not significantly different between the groups. Similar procedures were performed for patients with gastric cancer with and without CVMD. The median OS was 27.8 *versus* 26.8 *versus* 27.9 *versus* 28.3 months (*P *= 0.74) before matching and 28.7 *versus* 27.7 *versus* 34.2 *versus* 28.2 months (*P *= 0.81) after matching in gastric cancer patients without and with one, two, or multiple subtypes of CVMDs, respectively (Supplementary Figure 2). The survival time was not significantly different between the various groups.

**Figure 3:**
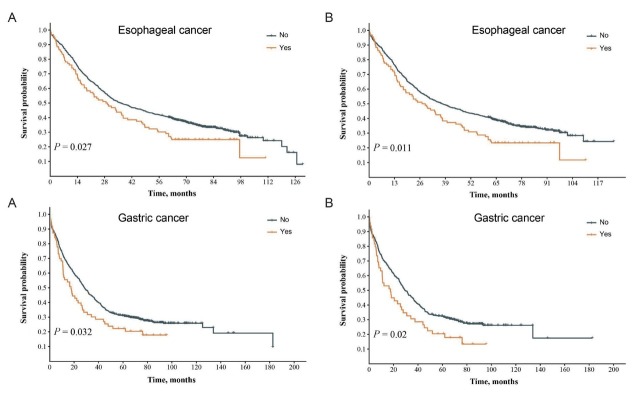
Survival time of esophageal and gastric cancer patients with or without ischemic heart disease. Kaplan–Meier survival plots of cancer patients with or without ischemic heart disease before (A) and after matching (B). *P* values were calculated using the log-rank test. “No” refers to the group of cancer patients without ischemic heart disease, and “Yes” refers to the group of cancer patients with ischemic heart disease.

All patients with esophageal and gastric cancer were further grouped according to the subtype of CVMD, including hypertension, diabetes, ischemic heart disease, and stroke. Before propensity score matching, the median OS was 36.5 and 29.1 months in esophageal cancer patients without and with ischemic heart disease (*P *= 0.027), respectively. After matching, the median OS was 37.4 and 27.9 months in esophageal cancer patients without and with ischemic heart disease (*P *= 0.011), respectively. In gastric cancer, the median OS was 28.4 and 17.5 months in patients without and with ischemic heart disease (*P *= 0.032) before matching, respectively. After matching, the median OS was 29.5 and 17.5 months in gastric cancer patients without and with ischemic heart disease, respectively, showing a significant difference (*P *= 0.02). The above results demonstrated that ischemic heart disease significantly reduced the OS in both esophageal and gastric cancer patients (Figure 3).

The median OS was 32.4 months in esophageal cancer patients with hypertension and 37.2 months in esophageal cancer patients without hypertension (*P *= 0.53) before matching. After matching, the median OS was 33 months in esophageal cancer patients with hypertension and 37.7 months in patients without hypertension (*P *= 0.62). Similar results were obtained for gastric cancer patients using the same method. The median OS was 27.9 and 27.1 months in patients with and without hypertension, respectively, before matching (*P *= 0.73). After matching, the median OS was 28.7 and 27.4 months in patients with and without hypertension, respectively (*P *= 0.87). These results suggest that hypertension does not affect the OS of patients with esophageal and gastric cancer (Supplementary Figure 3).

In esophageal cancer patients with and without diabetes, the median OS was 33.9 and 35.4 months, respectively, before matching (*P *= 0.4), and after matching, the median OS was 36.5 and 35.5 months, respectively (*P *= 0.64). In gastric cancer patients with and without diabetes, the median OS was 28.4 and 27.6 months before matching, respectively (*P *= 0.88). The median OS was 28.2 and 28.4 months in patients with and without diabetes, respectively, after matching (*P *= 0.47). No impact of diabetes on OS was found in patients with esophageal and gastric cancer (Supplementary Figure 4).

In esophageal cancer patients with and without stroke, the median OS was 34.1 and 35.3 months, respectively, before matching (*P *= 0.36) and 36.8 and 35.5 months, respectively, after matching (*P *= 0.55). In gastric cancer patients with and without stroke, the median OS was 36.2 and 27.1 months, respectively, before matching (*P *= 0.16) and 36.3 and 27.6 months, respectively, after matching (*P *= 0.097). There was no significant difference in the OS of esophageal and gastric cancer patients with and without stroke before and after matching (Supplementary Figure 5).

### Risk factors of esophageal and gastric cancer patients with ischemic heart disease, hypertension, diabetes, or stroke

A significant association was found between age, distant metastasis, tumor stage, and chemoradiotherapy and esophageal cancer patients with ischemic heart disease (all *P* < 0.05, Table 3). No significant association was found between sex, smoking, drinking, family history of tumor, recurrence, lymph node metastasis, or tumor differentiation and esophageal cancer patients with ischemic heart disease (all *P *> 0.05, Table 3).

**Table 3:** Association between clinical features and ischemic heart disease in esophageal cancer patients

**Characteristics**	**Patients without ischemic heart disease (*n* = 1109)**	**Patients with ischemic heart disease (*n* = 96)**	***P* value**
Sex			0.761
Male	676 (61.0)	57 (59.4)	
Female	433 (39.0)	39 (40.6)	
Age			< 0.001
≤ 60 years	334 (30.1)	9 (9.4)	
61–74 years	626 (56.5)	54 (56.2)	
≥ 75 years	149 (13.4)	33 (34.4)	
Smoking history			0.887
No	743 (67.0)	65 (67.7)	
Yes	366 (33.0)	31 (32.3)	
Drinking history			0.192
No	952 (85.8)	87 (90.6)	
Yes	157 (14.2)	9 (9.4)	
Family history of tumor			0.465
No	817 (73.7)	74 (77.1)	
Yes	292 (26.3)	22 (22.9)	
Recurrence during follow-up			0.667
No	1014 (91.4)	89 (92.7)	
Yes	95 (8.6)	7 (7.3)	
Lymph node metastasis			0.138
No	800 (72.1)	76 (79.2)	
Yes	309 (27.9)	20 (20.8)	
Distant metastasis			0.003
No	929 (83.8)	69 (71.9)	
Yes	180 (16.2)	27 (28.1)	
Tumor stage			0.004
I	473 (42.6)	41 (42.7)	
II	278 (25.1)	12 (12.5)	
III	178 (16.1)	16 (16.7)	
IV	180 (16.2)	27 (28.1)	
Tumor differentiation			0.214
Well differentiated	513 (46.2)	42 (43.8)	
Moderately differentiated	408 (36.8)	31 (32.2)	
Poorly differentiated	188 (17.0)	23 (24.0)	
Treatment regimen			0.001
Without chemo-radiotherapy	681 (61.4)	33 (34.4)	
With chemo-radiotherapy	428 (38.6)	63 (65.6)	

Data are presented as *n* (%).

A significant association was found between sex, age, lymph node metastasis, tumor differentiation, and chemoradiotherapy and gastric cancer patients with ischemic heart disease (all *P* < 0.05, Table 4). No significant association was found between smoking, drinking, family history of tumor, recurrence, distant metastasis, or tumor stage and gastric cancer patients with ischemic heart disease (all *P *> 0.05, Table 4). These results suggest that both esophageal and gastric cancer patients with ischemic heart disease share the risk factors of age and chemoradiotherapy.

**Table 4:** Association between clinical features and ischemic heart disease in gastric cancer patients

**Characteristics**	**Patients without ischemic heart disease (*n* = 806)**	**Patients with ischemic heart disease (*n* = 63)**	***P* value**
Sex			0.006
Male	613 (76.1)	38 (60.3)	
Female	193 (23.9)	25 (39.7)	
Age			< 0.001
≤ 60 years	359 (44.5)	12 (19.0)	
61–74 years	364 (45.2)	35 (55.6)	
≥ 75 years	83 (10.3)	16 (25.4)	
Smoking history			0.659
No	515 (63.9)	42 (66.7)	
Yes	291 (36.1)	21 (33.3)	
Drinking history			0.960
No	680 (84.4)	53 (84.1)	
Yes	126 (15.6)	10 (15.9)	
Family history of tumor			0.630
No	605 (75.1)	49 (77.8)	
Yes	201 (24.9)	14 (22.2)	
Recurrence during follow-up			0.613
No	748 (92.8)	60 (95.2)	
Yes	58 (7.2)	3 (4.8)	
Lymph node metastasis			0.018
No	400 (49.6)	41 (65.1)	
Yes	406 (50.4)	22 (34.9)	
Distant metastasis			0.249
No	782 (96.8)	63 (100)	
Yes	24 (3.0)	0	
Tumor stage			0.282
I	180 (22.3)	12 (19.1)	
II	309 (38.3)	22 (34.9)	
III	293 (36.4)	29 (46.0)	
IV	24 (3.0)	0	
Tumor differentiation			0.002
Well differentiated	102 (12.7)	17 (27.0)	
Moderately differentiated	412 (51.1)	33 (52.4)	
Poorly differentiated	292 (36.2)	13 (20.6)	
Treatment regimen			0.001
Without chemo-radiotherapy	647 (80.3)	38 (60.3)	
With chemo-radiotherapy	159 (19.7)	25 (39.7)	

Data are presented as *n* (%).

A significant association was found between sex, age, and smoking and esophageal cancer patients with hypertension (all *P* < 0.05, Supplementary Table 2). No significant association was found between other clinical factors and esophageal cancer patients with hypertension (all *P *> 0.05, Supplementary Table 2). The association was found to be significant between age, lymph node metastasis, and tumor differentiation and gastric cancer patients with hypertension (all *P* < 0.05, Supplementary Table 3). No significant association was found between other clinical factors and gastric cancer patients with hypertension (all *P *> 0.05, Supplementary Table 3). These results demonstrated that age was the only risk factor for both esophageal and gastric cancer patients with hypertension.

Smoking was the only factor significantly associated with esophageal cancer patients with diabetes (*P* < 0.05, Supplementary Table 4). No significant association was found between other clinical factors and esophageal cancer patients with diabetes (*P* > 0.05, Supplementary Table 4). Sex and age were significantly associated with gastric cancer patients with diabetes (*P* < 0.05, Supplementary Table 5). No significant association was found between other clinical factors and gastric cancer patients with diabetes (*P* > 0.05, Supplementary Table 5). No shared associated clinical factors were found between esophageal cancer and gastric cancer patients with diabetes.

A significant association was found between age, distant metastasis, and tumor stage and esophageal cancer patients with stroke (all *P* < 0.05, Supplementary Table 6). No significant association was found between other clinical factors and esophageal cancer patients with stroke (all *P *> 0.05; Supplementary Table 6). The association was found to be significant between age, tumor differentiation, and chemoradiotherapy and gastric cancer patients with stroke (all *P* < 0.05, Supplementary Table 7). No significant association was found between other clinical factors and gastric cancer patients with stroke (all *P *> 0.05; Supplementary Table 7). These results suggest that age is the only factor related to both esophageal and gastric cancer patients with stroke.

## Discussion

Metabolic syndrome is a cluster of morbidities including central obesity, insulin resistance, and dyslipidemia. It has been shown to increase the risk of cancer-related death and CVMDs.^[18, 19, 20]^ Accumulating evidence suggests a link between cancer and CVMDs, in addition to shared risk factors such as smoking, obesity, age, and inflammation.^[21,22]^ Cancer and related therapeutics may increase the risk of thrombosis and adverse cardiovascular events in this population.^[23, 24, 25]^ Thus, the risk factors for CVMDs are different in the general population and in patients with cancer. Other reports have suggested that CVMDs may increase the risk of cancer.^[22,26,27]^ As CVMD has been excluded from cancer studies in most clinical trials, the association between CVMDs and different cancers remains unclear.

In this study, a total of 2074 patients with esophageal and gastric cancers were included, among which 34.1% (411/1205) of esophageal cancer patients and 34.3% (298/869) of gastric cancer patients had comorbidity with CVMDs, suggesting a heavy burden of CVMDs in the cohort of esophageal and gastric cancer patients. The results demonstrated that the OS was significantly shorter in both esophageal and gastric cancer patients with ischemic heart disease than in those without ischemic heart disease. These results indicate that it is necessary to prevent and treat ischemic heart disease during cancer treatment. The OS was not significantly different in esophageal and gastric cancer patients with or without hypertension, diabetes, and stroke. In this study, patients were followed up for a median of 79.8 and 79.3 months for esophageal and gastric cancers, respectively. Increasing the length of follow-up may lead to different conclusion for cancer patients with comorbidities of hypertension, stroke, or diabetes.

Further analysis of the association of clinical factors with esophageal and gastric cancer patients with ischemic heart disease, hypertension, diabetes, and stroke is required. An association was frequently found between each clinical factor and esophageal or gastric cancer patients with different diseases, including ischemic heart disease, hypertension, diabetes, and stroke; however, shared risk factors were seldom found in both esophageal and gastric cancer patients with other diseases. Our results demonstrated that old age and chemoradiotherapy are shared risk factors for both esophageal and gastric cancer patients with ischemic heart disease. Many studies have confirmed that chemoradiotherapy causes ischemic heart disease, and the incidence of ischemic heart disease increases with age. Age is the only shared risk factor for both esophageal and gastric cancer patients with hypertension or stroke. No shared risk factors were found for esophageal and gastric cancer patients with diabetes. Mortality risk was different in cancer patients with CVMD and in those with CVMD among the general population. Cancer itself and its treatment may increase the risk of mortality in patients with CVMD. The etiology and mechanisms of CVMD and cancer are complex. Similar risk factors, including smoking, obesity, and age, have been frequently reported in cancer patients and patients with atrial fibrillation, atherosclerosis, stroke, myocardial infarction, and hypertension. Recent evidence suggests that inflammation is the mutual pathophysiologic link between atherothrombosis and cancer; atherosclerosis and cancer may influence each other during progression.^[21,28]^ An increased risk of both cancer and cardiovascular disease was found in rheumatoid arthritis, which is a high-level inflammatory disease, and anti-inflammatory therapies have shown promise in reducing cardiovascular events and cancer risk.^[29,30]^ Canakinumab, an interleukin (IL)-1β inhibitor, significantly reduced cardiovascular-related death.^[31,32]^ IL-1 mediates tumor angiogenesis, metastasis, and immune evasion.^[33,34]^ The application of IL-6 and IL-1 antagonists increased the survival time of cancer patients in clinical trials^[21]^ and reduced lung cancer mortality.^[35]^ Inflammation may also lead to atherosclerosis and cancer via the production of reactive oxygen species.^[36]^

Cancer itself may increase the risk of thrombotic events, both arterial and venous.^[23]^ The incidence of ischemic cardiovascular events varies among different types of cancers,^[37]^ and treatment with various regimens may alter the thrombotic risk in different cancers.^[21]^ Radiation may directly increase the risk of ischemic heart disease.^[38, 39, 40]^ Cytotoxic drugs also cause cardiotoxicity and vascular toxicity, increasing atherosclerotic risk by damaging endothelial cells and plaque erosion.^[41]^ Immune checkpoint inhibitors are novel therapeutic agents that have been reported to increase the risk of vascular events;^[42]^ recent evidence suggests that immune checkpoint inhibitors increase the risk of cardiovascular events by threefold.^[43]^

In summary, it is reasonable to deduce that cancer itself, radiotherapy, and chemotherapy may cause inflammation, atherosclerosis, DNA damage, and cardiovascular thrombotic events, which generate or promote ischemic heart disease and death. Our results suggest that both esophageal and gastric cancer patients with ischemic heart disease have significantly reduced OS compared to those without ischemic heart disease. It is important to prevent and manage ischemic heart disease during the treatment of patients with esophageal and gastric cancer.

The limitations of this retrospective study were inevitable. For some patients, it was difficult to determine the specific cause of death without advanced laboratory testing. In addition, data on the potential confounding factors, including obesity, body mass index, and tumor location, were lacking. To further understand the interaction between cancer and CVMD, a large-scale, multicenter, prospective study is needed.

In conclusion, ischemic heart disease significantly reduced the survival time of esophageal and gastric cancer patients in Xinxiang, a high-incidence area of esophageal and gastric cancer. In this cohort, an effect of hypertension, diabetes, and stroke on the long-term survival of patients with esophageal and gastric cancers was not found.

## Supplementary Material

Supplementary Materi
