# Supplementary material for: Long-term outcomes of esophageal and gastric cancer patients with cardiovascular and metabolic diseases: A two-center propensity score-matched cohort study
Source: J Transl Int Med. 2023 Sep 2;11(3):234–45. doi: 10.2478/jtim-2023-0112 (PMC10561076; doi:10.2478/jtim-2023-0112)
Supplement: Supplementary file 1 — Supplementary Materi [file jtim-2023-0112_sm.pdf]

Supplementary Material

Supplementary Table 1: Constitution of CVMD in esophageal and gastric cancer patients

Characteristics	Constitution of CVMD in esophageal cancer patients (*n* = 1205)	Constitution of CVMD in gastric cancer patients (*n* = 869)
Presence of CVMD		
No	794 (65.9)	571 (65.7)
Yes	411 (34.1)	298 (34.3)
Number of CVMDs		
0	794 (65.9)	571 (65.7)
1	293 (24.3)	215 (24.7)
2	99 (8.2)	65 (7.5)
> 2	19 (1.6)	18 (2.1)
Hypertension		
No	932 (77.3)	656 (75.5)
Yes	273 (22.7)	213 (24.5)
Diabetes		
No	1107 (91.9)	810 (93.2)
Yes	98 (8.1)	59 (6.8)
Ischemic heart disease		
No	1109 (92.0)	806 (92.8)
Yes	96 (8.0)	63 (7.2)
Stroke		
No	1122 (93.1)	804 (92.5)
Yes	83 (6.9)	65 (7.5)

Data are presented as *n* (%). CVMDs: cardiovascular and metabolic diseases.

Supplementary Table 2: Association between clinical features and hypertension in esophageal cancer patients

Characteristics	Patients without hypertension (*n* = 932)	Patients with hypertension (*n* = 273)	*P* value
Sex			0.005
Male	587 (63.0)	146 (53.5)	
Female	345 (37.0)	127 (46.5)	
Age			
≤ 60 years	287 (30.8)	56 (20.5)	0.004
60–75 years	511 (54.8)	169 (61.9)	
≥ 75 years	134 (14.4)	48 (17.6)	
Smoking history			0.001
No	602 (64.6)	206 (75.5)	
Yes	330 (35.4)	67 (24.5)	
Drinking history			0.357
No	799 (85.7)	240 (87.9)	
Yes	133 (14.3)	33 (12.1)	
Family history of tumor			0.112
No	679 (72.9)	212 (77.7)	
Yes	253 (27.1)	61 (22.3)	
Recurrence during follow-up			0.602
No	851 (91.3)	252 (92.3)	
Yes	81 (8.7)	21 (7.7)	
Lymph node metastasis			0.483
No	673 (72.2)	203 (74.4)	
Yes	259 (27.8)	70 (25.6)	
Distant metastasis			0.266
No	778 (83.5)	220 (80.6)	
Yes	154 (16.5)	53 (19.4)	
Tumor stage			0.584
I	398 (42.7)	116 (42.5)	
II	231 (24.8)	59 (21.6)	

Supplementary Table 2: Association between clinical features and hypertension in esophageal cancer patients

Characteristics	Patients without hypertension (*n* = 932)	Patients with hypertension (*n* = 273)	*P* value
III	149 (16.0)	45 (16.5)	
IV	154 (16.5)	53 (19.4)	
Tumor differentiation			0.385
Well differentiated	438 (47.0)	117 (42.9)	
Moderately differentiated	337 (36.2)	102 (37.4)	
Poorly differentiated	157 (16.8)	54 (19.7)	
Treatment regimen			0.277
Without chemo-radiotherapy	560 (60.1)	154 (56.4)	
With chemo-radiotherapy	372 (39.9)	119 (43.6)	

Data are presented as *n* (%).

Supplementary Table 3: Association between clinical features and hypertension in gastric cancer patients

Characteristics	Patients without hypertension (*n* = 656)	Patients with hypertension (*n* = 213)	*P* value
Sex			0.055
Male	502 (76.5)	149 (70.0)	
Female	154 (23.5)	64 (30.0)	
Age (years)			<0.001
≤ 60	317 (48.3)	54 (25.4)	
60–75	275 (41.9)	124 (58.2)	
≥ 75	64 (9.8)	35 (16.4)	
Smoking history			0.678
No	423 (64.5)	134 (62.9)	
Yes	233 (35.5)	79 (37.1)	
Drinking history			0.219
No	559 (85.2)	174 (81.7)	
Yes	97 (14.8)	39 (18.3)	
Family history of tumor			0.221
No	487 (74.2)	167 (78.4)	
Yes	169 (25.8)	46 (21.6)	
Recurrence during follow-up			0.362
No	607 (92.5)	201 (94.4)	
Yes	49 (7.5)	12 (5.6)	
Lymph node metastasis			0.028
No	319 (48.6)	122 (57.3)	
Yes	337 (51.4)	91 (42.7)	
Distant metastasis			0.955
No	638 (97.3)	207 (97.2)	
Yes	18 (2.7)	6 (2.8)	
Tumor stage			0.073
I	135 (20.6)	57 (26.8)	
II	265 (40.4)	66 (31.0)	
III	238 (36.3)	84 (39.4)	
IV	18 (2.7)	6 (2.8)	
Tumor differentiation			<0.001
Well differentiated	80 (12.2)	39 (18.3)	
Moderately differentiated	321 (48.9)	124 (58.2)	
Poorly differentiated	255 (38.9)	50 (23.5)	
Treatment regimen			0.086
Without chemo-radiotherapy	526 (80.2)	159 (74.6)	
With chemo-radiotherapy	130 (19.8)	54 (25.4)	

Data are presented as *n* (%).

Supplementary Table 4: Association between clinical features and diabetes in esophageal cancer patients

Characteristics	Patients without diabetes (*n* = 1107)	Patients with diabetes (*n* = 98)	*P* value
Sex			0.153
Male	680 (61.4)	53 (54.1)	
Female	427 (38.6)	45 (45.9)	
Age			0.069
≤ 60 years	325 (29.4)	18 (18.4)	
60–75 years	617 (55.7)	63 (64.3)	
≥ 75 years	165 (14.9)	17 (17.3)	
Smoking history			0.037
No	733 (66.2)	75 (76.5)	
Yes	374 (33.8)	23 (23.5)	
Drinking history			0.878
No	954 (86.2)	85 (86.7)	
Yes	153 (13.8)	13 (13.3)	
Family history of tumor			0.070
No	811 (73.3)	80 (81.6)	
Yes	296 (26.7)	18 (18.4)	
Recurrence during follow-up			0.790
No	1014 (91.6)	89 (90.8)	
Yes	93 (8.4)	9 (9.2)	
Lymph node metastasis			0.678
No	803 (72.5)	73 (74.5)	
Yes	304 (27.5)	25 (25.5)	
Distant metastasis			0.545
No	919 (83.0)	79 (80.6)	
Yes	188 (17.0)	19 (19.4)	
Tumor stage			0.484
I	468 (42.3)	46 (47.0)	
II	268 (24.2)	22 (22.4)	
III	183 (16.5)	11 (11.2)	
IV	188 (17.0)	19 (19.4)	
Tumor differentiation			0.873
Well differentiated	512 (46.3)	43 (43.9)	
Moderately differentiated	401 (36.2)	38 (38.8)	
Poorly differentiated	194 (17.5)	17 (17.3)	
Treatment regimen			0.052
Without chemo-radiotherapy	665 (60.1)	49 (50.0)	
With chemo-radiotherapy	442 (39.9)	49 (50.0)	

Data are presented as *n* (%).

Supplementary Table 5: Association between clinical features and diabetes in gastric cancer patients

Characteristics	Patients without diabetes (*n* = 810)	Patients with diabetes (*n* = 59)	*P* value
Sex			0.025
Male	614 (75.8)	37 (62.7)	
Female	196 (24.2)	22 (37.3)	
Age			0.034
≤ 60 years	355 (43.8)	16 (27.1)	
60–75 years	363 (44.8)	36 (61)	
≥ 75 years	92 (11.4)	7 (11.9)	
Smoking history			0.283
No	523 (64.6)	34 (57.6)	
Yes	287 (35.4)	25 (42.4)	
Drinking history			0.931
No	683 (84.3)	50 (84.7)	
Yes	127 (15.7)	9 (15.3)	

Supplementary Table 5: Association between clinical features and diabetes in gastric cancer patients

Characteristics	Patients without diabetes (*n* = 810)	Patients with diabetes (*n* = 59)	*P* value
Family history of tumor			0.288
No	613 (75.7)	41 (69.5)	
Yes	197 (24.3)	18 (30.5)	
Recurrence during follow-up			0.115
No	750 (92.6)	58 (98.3)	
Yes	60 (7.4)	1 (1.7)	
Lymph node metastasis			0.274
No	407 (50.2)	34 (57.6)	
Yes	403 (49.8)	25 (42.4)	
Distant metastasis			1.000
No	787 (97.2)	58 (98.3)	
Yes	23 (2.8)	1 (1.7)	
Tumor stage			0.771
I	176 (21.7)	16 (27.1)	
II	310 (38.3)	21 (35.6)	
III	301 (37.2)	21 (35.6)	
IV	23 (2.8)	1 (1.7)	
Tumor differentiation			0.059
Well differentiated	105 (13.0)	14 (23.7)	
Moderately differentiated	420 (51.9)	25 (42.4)	
Poorly differentiated	285 (35.2)	20 (33.9)	
Treatment regimen			0.867
Without chemo-radiotherapy	639 (78.9)	46 (78.0)	
With chemo-radiotherapy	171 (21.1)	13 (22.0)	

Data are presented as *n* (%).

Supplementary Table 6: Association between clinical features and stroke in esophageal cancer patients

Characteristics	Patients without stroke (*n* = 1122)	Patients with stroke (*n* = 83)	*P* value
Sex			0.725
Male	681 (60.7)	52 (62.7)	
Female	441 (39.3)	31 (37.3)	
Age			0.003
≤ 60 years	332 (29.6)	11 (13.3)	
60–75 years	627 (55.9)	53 (63.9)	
≥ 75 years	163 (14.5)	19 (22.9)	
Smoking history			0.570
No	750 (66.8)	58 (69.9)	
Yes	372 (33.2)	25 (30.1)	
Drinking history			0.132
No	972 (86.6)	67 (80.7)	
Yes	150 (13.4)	16 (19.3)	
Family history of tumor			0.257
No	834 (74.3)	57 (68.7)	
Yes	288 (25.7)	26 (31.3)	
Recurrence during follow-up			0.100
No	1023 (91.2)	80 (96.4)	
Yes	99 (8.8)	3 (3.6)	
Lymph node metastasis			0.497
No	813 (72.5)	63 (75.9)	
Yes	309 (27.5)	20 (24.1)	
Distant metastasis			0.020
No	937 (83.5)	61 (73.5)	
Yes	185 (16.5)	22 (26.5)	

Supplementary Table 6: Association between clinical features and stroke in esophageal cancer patients

Characteristics	Patients without stroke (*n* = 1122)	Patients with stroke (*n* = 83)	*P* value
Tumor stage			0.041
I	476 (42.4)	38 (45.8)	
II	277 (24.7)	13 (15.7)	
III	184 (16.4)	10 (12.0)	
IV	185 (16.5)	22 (26.5)	
Tumor differentiation			0.168
Well differentiated	521 (46.4)	34 (41.0)	
Moderately differentiated	401 (35.7)	38 (45.8)	
Poorly differentiated	200 (17.9)	11 (13.2)	
Treatment regimen			0.058
Without chemo-radiotherapy	673 (60.0)	41 (49.4)	
With chemo-radiotherapy	449 (40.0)	42 (50.6)	

Data are presented as *n* (%).

Supplementary Table 7: Association between clinical features and stroke in gastric cancer patients

Characteristics	Patients without stroke (*n* = 804)	Patients with stroke (*n* = 65)	*P* value
Sex			0.927
Male	602 (64.9)	49 (75.4)	
Female	202 (25.1)	16 (24.6)	
Age			0.001
≤ 60 years	357 (44.4)	14 (21.5)	
60–75 years	357 (44.4)	42 (64.6)	
≥ 75 years	90 (11.2)	9 (13.9)	
Smoking history			0.530
No	513 (63.8)	44 (67.7)	
Yes	291 (36.2)	21 (32.3)	
Drinking history			0.951
No	678 (84.3)	55 (84.6)	
Yes	126 (15.7)	10 (15.4)	
Family history of tumor			0.981
No	605 (75.2)	49 (75.4)	
Yes	199 (24.8)	16 (24.6)	
Recurrence during follow-up			0.825
No	748 (93.0)	60 (92.3)	
Yes	56 (7.0)	5 (7.7)	
Lymph node metastasis			0.196
No	403 (50.1)	38 (58.5)	
Yes	401 (49.9)	27 (41.5)	
Distant metastasis			0.097
No	784 (97.5)	61 (93.8)	
Yes	20 (2.5)	4 (6.2)	
Tumor stage			0.186
I	174 (21.6)	18 (27.7)	
II	308 (38.3)	23 (35.4)	
III	302 (37.6)	20 (30.8)	
IV	20 (2.5)	4 (6.2)	
Tumor differentiation			< 0.001
Well differentiated	97 (12.1)	22 (33.8)	
Moderately differentiated	410 (51.0)	35 (53.8)	
Poorly differentiated	297 (36.9)	8 (12.4)	
Treatment regimen			0.022
Without chemo-radiotherapy	641 (79.7)	44 (67.7)	
With chemo-radiotherapy	163 (20.3)	21 (32.3)	

Data are presented as *n* (%).

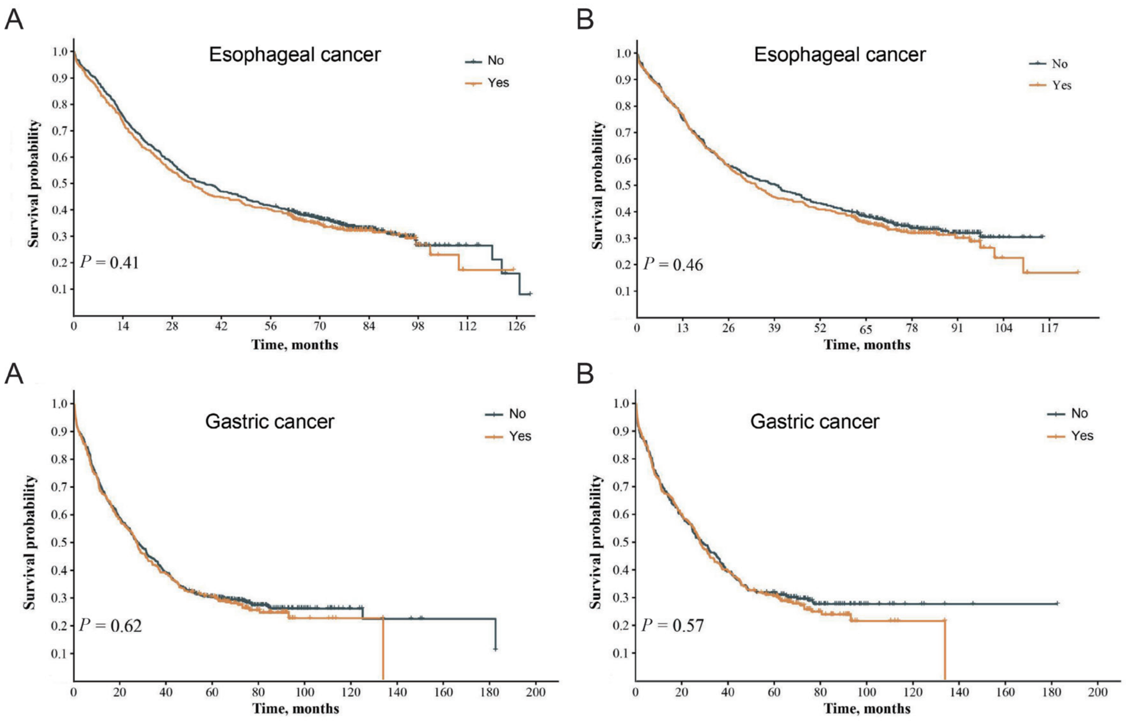

Supplementary Figure 1: Survival time of patients with esophageal and gastric cancer with or without CVMD. Kaplan–Meier survival plots of esophageal and gastric cancer patients with or without CVMD before (A) and after matching (B). *P* values were calculated using the log-rank test. “No” refers to the group of cancer patients without CVMD, and “Yes” refers to the group of cancer patients with CVMD. CVMDs: cardiovascular and metabolic diseases.

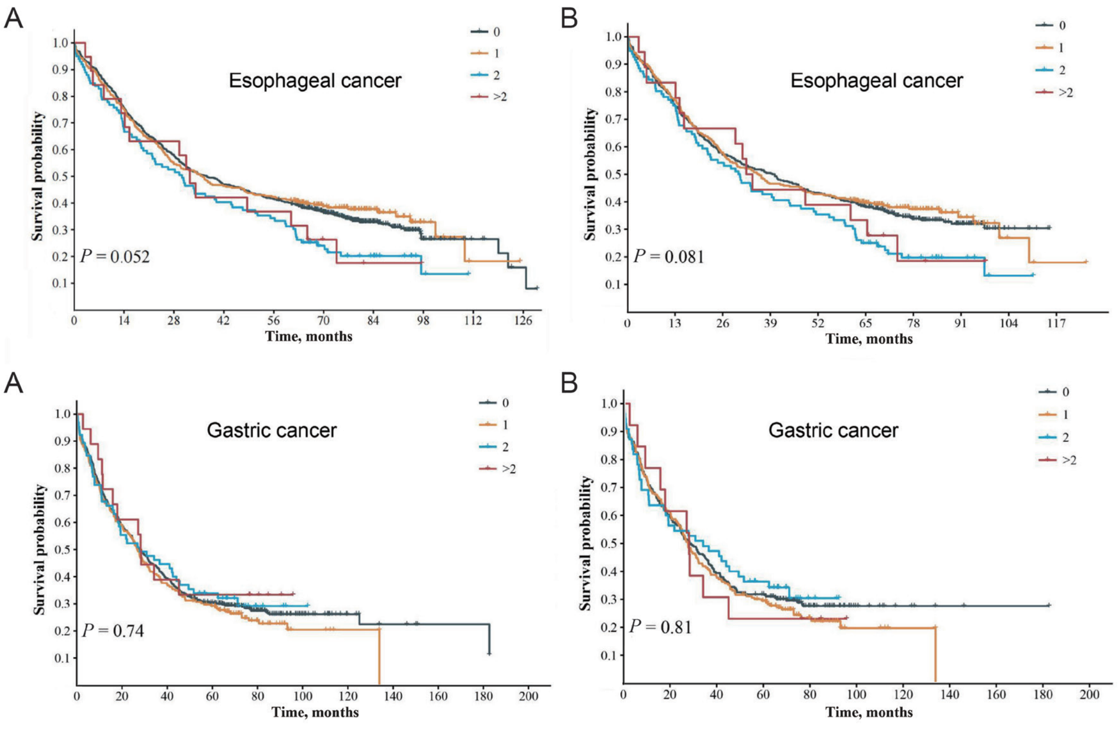

Supplementary Figure 2. Survival time of esophageal and gastric cancer patients with various subtypes of CVMDs. Kaplan–Meier survival plots of esophageal and gastric cancer patients with various subtypes of CVMDs before (A) and after matching (B). *P* values were calculated using the log-rank test. “0” refers to the group of cancer patients without CVMD, “1” refers to the group of cancer patients with one subtype of CVMD, “2” refers to the group of cancer patients with two subtypes of CVMD, “>2” refers to the group of cancer patients with multiple subtypes of CVMD. CVMDs: cardiovascular and metabolic diseases.

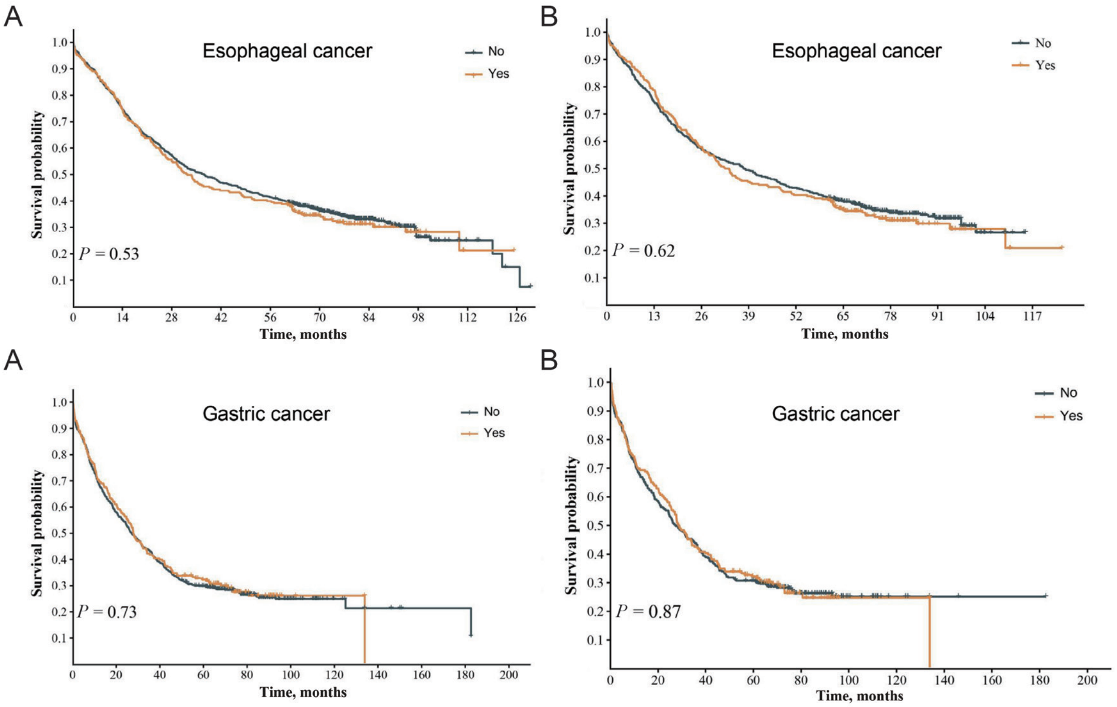

Supplementary Figure 3: Survival time of patients with esophageal and gastric cancer with or without hypertension. Kaplan–Meier survival plots of esophageal and gastric cancer patients with or without hypertension before (A) and after matching (B). *P* values were calculated using the log-rank test. "No" refers to the group of cancer patients without hypertension, and "Yes" refers to the group of cancer patients with hypertension.

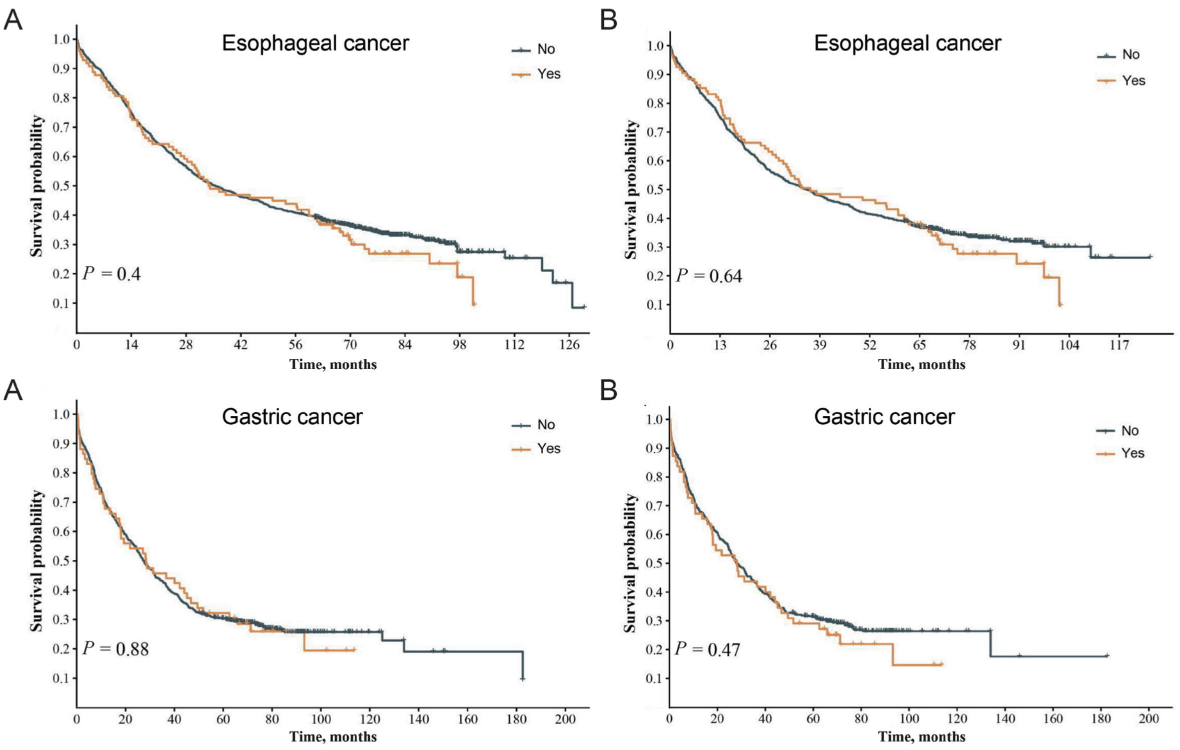

Supplementary Figure 4: Survival time of esophageal and gastric cancer patients with or without diabetes. Kaplan–Meier survival plots of esophageal and gastric cancer patients with or without diabetes before (A) and after matching (B). *P* values were calculated using the log-rank test. "No" refers to the group of cancer patients without diabetes, and "Yes" refers to the group of cancer patients with diabetes.

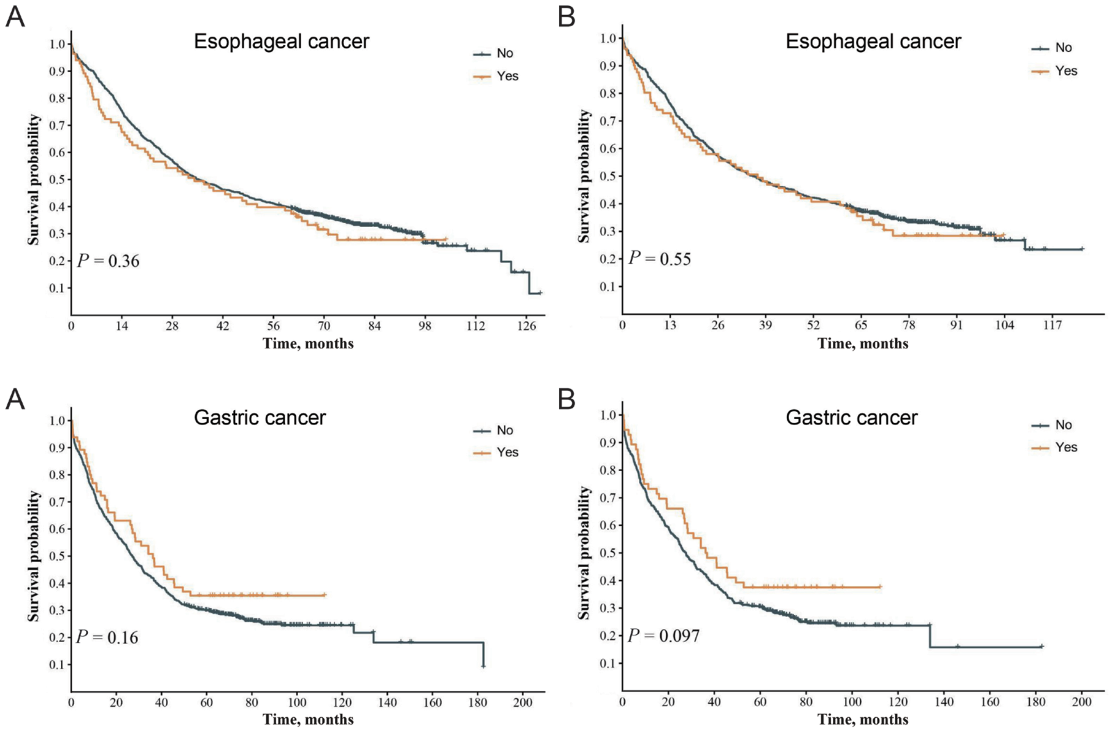

Supplementary Figure 5: Survival time of esophageal and gastric cancer patients with or without stroke. Kaplan–Meier survival plots of esophageal and gastric cancer patients with or without stroke before (A) and after matching (B). *P* values were calculated using the log-rank test. "No" refers to the group of cancer patients without stroke, and "Yes" refers to the group of cancer patients with stroke.
